# Self-Regulation and Psychopathology in Young Children

**DOI:** 10.1007/s10578-022-01322-x

**Published:** 2022-02-11

**Authors:** Jamie M. Lawler, Jerrica Pitzen, Kristin Aho, Ka I Ip, Yanni Liu, Jessica Hruschak, Maria Muzik, Katherine L. Rosenblum, Kate D. Fitzgerald

**Affiliations:** 1 Eastern Michigan University, Department of Psychology, Ypsilanti, MI; 2 Yale University, New Haven, CT; 3 University of Michigan, Department of Psychiatry, Ann Arbor, MI; 4 Wayne State University, Detroit, MI

**Keywords:** self-regulation, internalizing, externalizing, inhibitory control, attention

## Abstract

The current study examined concurrent relationships between children’s self-regulation, measured behaviorally and by parent-report, and children’s internalizing and externalizing symptoms. The aim was to distinguish which components of self-regulation (attention vs. inhibitory control, “hot” vs. “cool” regulation) best predict dimensional symptomatology and clinical disorders in young children. The participants were 120 children, ages 4-8 years old. Results showed that greater parent-reported attention was associated with fewer internalizing *and* externalizing symptoms. Behaviorally-measured hot inhibitory control related to fewer internalizing symptoms, whereas parent-reported inhibitory control related to fewer externalizing symptoms. Similar patterns emerged for clinical diagnoses, with parent-rated attention most strongly predicting disorders across domains. Results support prior evidence implicating self-regulatory deficits in externalizing problems, while also demonstrating that components of self-regulation are impaired with internalizing symptoms. Further, different sub-components of self-regulation relate to different dimensions of psychopathology in children. Interventions should target these areas in children at-risk for disorders.

## Summary

The aim of the current study was to examine the association between self-regulation and internalizing and externalizing symptoms in young children. In particular, we sought to determine whether the subcomponents of attention vs. inhibitory control, as well as hot vs. cool systems would be stronger predictors of internalizing and externalizing symptoms and disorders when measured behaviorally and by parent-report. The participants in the study were 120 children, ages 4-8 years old, oversampled for those at risk for psychopathology in order to yield a wide range of self-regulation abilities and anxiety/depression risk, consistent with a Research Domain Criteria approach. Hot inhibitory control was measured with a delay of gratification task, while cool inhibitory control was measured with a computerized go/no-go task. Parents reported on their child’s attention and inhibitory control. Results showed that greater parent-reported attention and behaviorally-measured hot inhibitory control relate to fewer internalizing symptoms, while parent ratings of both attention and inhibitory control were most strongly associated with fewer externalizing symptoms. Anticipation of the reward (attention on the treat) during the delay of gratification task predicted fewer internalizing symptoms and lower risk of disorder, as well as fewer externalizing symptoms at a trend level. Similar patterns emerged for clinical diagnoses, with parent-rated attention most strongly predicting disorders across domains. Cool inhibitory control did not relate to either internalizing or externalizing symptoms or disorders. Results support prior evidence implicating self-regulatory deficits in externalizing problems, while also demonstrating that components of self-regulation are impaired with internalizing symptoms. Low levels of parent-reported attention generalized across symptom domains. By contrast, specific deficits of behaviorally indexed “hot” self-regulation with internalizing severity and of parent-rated inhibitory control with externalizing symptoms, suggests that different sub-components of self-regulatory control may relate to different dimensions of psychopathology in children. Interventions should target these areas to promote resilience in children at risk for disorders. Implementing interventions that improve children’s ability to inhibit behavior and focus attention could potentially prevent the development of symptoms in young children.

Extensive research over the past several decades has demonstrated robust relationships between childhood self-regulation and numerous developmental outcomes, including academic achievement, mental and physical health, and financial wellbeing into adulthood [1–3]. Self-regulation refers to a child’s developing ability to control their thoughts, behaviors, and emotions. Self-regulation has been reliably associated with fewer externalizing behavior problems in children (see [4] for review), however its relationship with internalizing symptoms has been less well established. Also still unclear is whether distinct aspects of self-regulation (e.g. inhibitory control vs. attention, hot vs. cool) are differentially associated with externalizing and internalizing symptomatology. The Research Domain Criteria (RDoC; [5]) emphasize the importance of elucidating mechanistic pathways towards externalizing and internalizing symptoms, across a normal to abnormal range, based on psychologically relevant constructs indexed at varying levels of analysis. Thus, it is crucial to understand how self-regulation, indexed using different units of analysis (e.g., objective behavior, parent-report on child) may contribute to the development of clinical disorders in addition to dimensional symptomatology in early life. The purpose of the current study was to examine the associations between several different aspects of self-regulation and internalizing and externalizing symptoms, including clinically significant disorders, in 4- to 8-year-old children.

## Self-Regulation

There are various ways of categorizing aspects of self-regulation. First, researchers have identified inhibitory and attentional control as interrelated but separable components of self-regulation. Inhibitory control refers to the ability to inhibit an automatic or prepotent response, while attentional control involves attention focus (the ability to focus on targeted stimuli in the environment while ignoring extraneous stimuli) and attention shifting (the ability to flexibly shift attention as a task demands; [6]). While related, attention and inhibitory control have different developmental trajectories and correlates [7]. For example, Eisenberg and colleagues [8] found that attention but not inhibitory control was protective against internalizing symptoms; however, in other samples inhibitory control has also been found to be protective [9]. Across studies, attentional control has been more robustly associated with fewer internalizing symptoms than inhibitory control [10–14]. In fact, inhibitory control has at times been found to be *positively* related to certain types of internalizing symptoms, especially among behavioral inhibited children [13, 15]. By contrast, deficits of inhibitory control have been particularly related to externalizing symptoms, including attention deficit hyperactivity disorder (ADHD) [16–19]. The current study will test for convergent and divergent associations between the inhibitory and attentional control components of self-regulation and child symptomatology across internalizing and externalizing domains.

**Figure 1:**
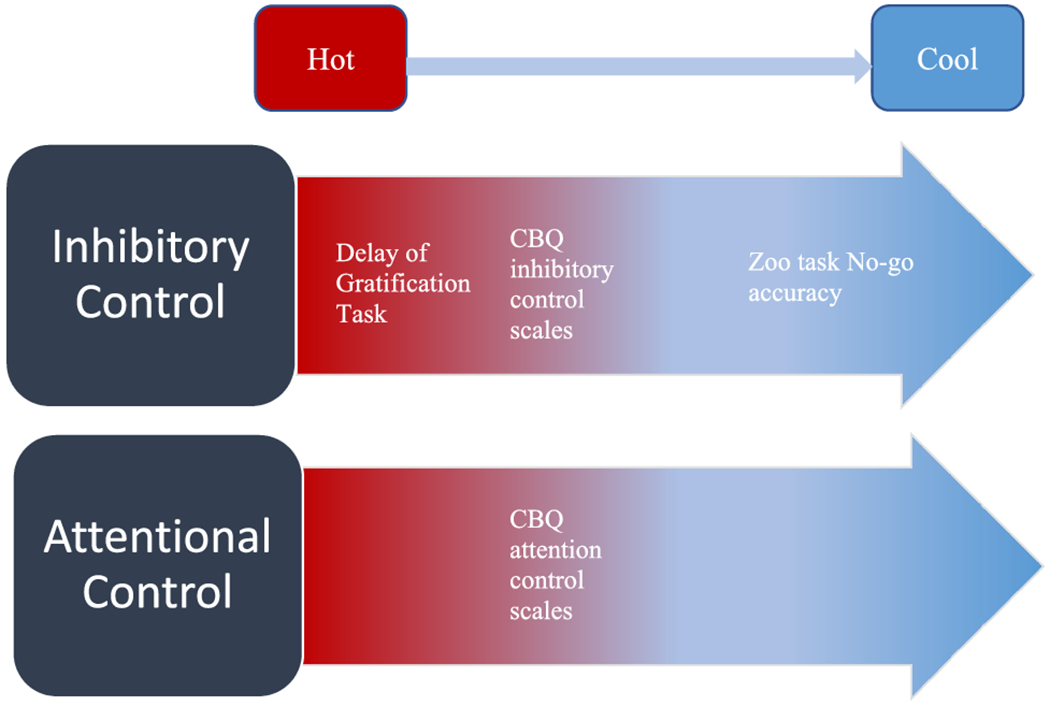
Inhibitory and attentional control can be conceptualized as separable components of self-regulatory control. Both components can be considered along a hot-to-cool spectrum in which hot self-regulation (red) occurs during emotion-laden tasks, whereas cool self-regulation (blue) is more strictly cognitive in nature. For the present study, inhibitory control was indexed across the hot to cool spectrum using behavioral and parent-report measures, whereas attentional control was indexed only at mid-spectrum with parent-report (see tasks/measures listed in white).

A second way to understand self-regulation distinguishes between “hot” self-regulation that occurs under emotion laden contexts, versus “cool” regulation that does not involve strong emotions or salient rewards and can be considered more strictly cognitive in nature [20, 21]. These systems of hot and cool self-regulation span across the subcomponents of attention and inhibitory control, meaning that measures of attention and inhibitory control can be either hot or cool, or a hot or cool task may require both attention and inhibitory control. Hot and cool systems can be thought of as opposing ends of a linear spectrum, in contrast to attention and inhibitory control which are not considered as such (see Figure 1). The distinction between hot and cool self-regulation has garnered increasing attention in the recent years as researchers have found important differences between these systems in relation to child characteristics such as academic performance and psychopathology [22–24]. Kim and colleagues [25] found that hot (emotion-laden) regulation was associated with behavior problems (combined internalizing and externalizing) while cool (cognitive) regulation was associated with academic outcomes. Backer-Grøndahl and colleagues [26] found hot regulation specifically predicted externalizing problems, while neither hot nor cool regulation predicted internalizing problems.

A child’s behavior in a task may also shift how hot or cool the task is. For example, past research using delay of gratification paradigms has shown that excessive anticipation of the reward leads to decreased success in delaying and to increased emotional distress (making the task more “hot”), whereas turning attention away from the reward leads to improved delay time (and potentially makes the task more “cool”) [27, 28]. No prior study to our knowledge has examined the associations between anticipatory behavior (attentional focus on the treat) in a reward delay task and child symptomatology.

## Units of Self-regulation Measurement

The RDoC initiative emphasizes the need for integrating many levels of information to understand the nature of mental health and illness. Two important units of analysis for measuring self-regulation in children are the behavioral level and the self-/parent-report level. In general, available parent-report measures of self-regulation differentiate between attention and inhibitory control, but do not differentiate hot vs. cool regulation and tend toward capturing hot regulation required for everyday contexts. Thus, behavioral tasks are especially important for distinguishing between hot and cool self-regulation [29]. A meta-analysis showed moderate convergence between parent-report and behavioral tasks, but noted substantial heterogeneity, leading the authors to conclude that self-regulation is a coherent but multidimensional construct that is best assessed using multiple methods [30]. Behavioral delay tasks (mostly hot) were more strongly associated with parent-report questionnaires than behavioral tests of executive function (mostly cool). Further, type of measurement matters for associations with psychopathology. For example, depression was associated with poor inhibitory control as rated by parents (more hot), but the reverse was true on a behavioral measure of executive function (more cool): adolescents high in depression showed better inhibitory control than youth not experiencing symptoms on a cool computerized response inhibition task [31].

Despite these hot-cool distinctions between parent-reported and behaviorally-indexed self-regulation in predicting psychopathology, many studies use only parent-report measures of self-regulation (e.g. [32]) or combine parent-report and child performance on behavioral tests into a singular self-regulation construct (e.g. [33]). A recent meta-analysis that did not differentiate between subcomponents or hot/cool systems of self-regulation but did examine the method of measurement, found evidence that behaviorally measured self-regulation predicted more strongly to externalizing symptoms, while parent-report predicted more strongly to internalizing symptoms [3].

## Measurement of Psychopathology

The majority of studies examine the relationship between self-regulation and symptomatology along a spectrum in community samples (e.g. [34, 35]), while few studies examine what elements contribute to clinically significant disorders. RDoC posits that dimensions of behavior (e.g., self-regulation), measured across units of analysis, will relate to psychopathology across the full spectrum of severity, but additional work is needed to determine whether certain aspects of self-regulation are differentially associated with clinically significant symptomatology (e.g., presence or absence of a disorder) compared with dimensionally-measured symptoms across a normal to abnormal range (e.g. [36]). Clarification of the relation of early childhood internalizing and externalizing symptoms with self-regulation –indexed behaviorally and by parent-report, across subcomponents and the hot/cool system– is needed. Such work is especially important given the risk that sub-clinical symptoms and “disorder” in early childhood poses for subsequent social and emotional maladjustment in later life (e.g. [37, 38]).

## Current Study

Cumulative results suggest it is important to measure self-regulation in multiple ways and examine the associations of different domains with symptomatology independently. However, many unanswered questions remain regarding the strongest predictors of outcomes. Thus, the current study examined concurrent relationships between children’s self-regulation, measured by behavioral tasks and parent-report, and children’s internalizing and externalizing symptomatology. The aim was to distinguish which components of self-regulation best predict psychopathology in young children. We examined inhibitory control vs. attentional control by comparing parent-report on typical child actions and reactions in these domains. Guided by our review and synthesis of prior findings (e.g., 8-19), we hypothesized that the parent-reported attentional sub-component of self-regulation would be more highly associated with internalizing symptoms, while both parent-reported and behaviorally measured inhibitory control would be more associated with externalizing symptoms. We also explored the possibility that parent-reported attentional and inhibitory control may differentially associate with psychopathology when examining clinically significant disorders compared to dimensional symptom counts. Further, we examined hot vs. cool self-regulation systems using behavioral tasks. Cool inhibitory control was measured on a computerized go/no-go task and hot inhibitory control was measured on a delay of gratification task. Consistent with prior literature (e.g. [25]), we predicted that hot inhibitory control would be more associated with both internalizing and externalizing symptomatology than the cool inhibitory control.

## Methods

### Participants

The participants in this study were 120 4-8 year old children (55% female), oversampled for those at risk for psychopathology in order to yield a wide range of self-regulation abilities and anxiety/depression risk, consistent with a Research Domain Criteria approach [5]. Participants were part of a broader project (N=140) examining behavioral and neurobiological markers of childhood depression and anxiety symptoms and were recruited to represent a continuum of low to high presence of psychopathology. The 20 omitted participants took part in the study before several of the current measures were added, and thus were excluded from analyses. Participants were recruited from outpatient psychiatry clinics (n=7 participants), a prior study of pregnant women at risk for anxiety (n=43), pediatrician’s offices (n=26), online recruitment for clinical studies (n=23), and local schools/community fliers/word of mouth (n=21). Children positive for neurodevelopmental delay, serious medical condition, or autism were screened out.

Participants were 6.0 years old on average (SD =1.2 years) at testing. Children were primarily Caucasian (65.8%), followed by more than one race (19.2%), African American (10%), Asian or Pacific Islander (2.5%), Latino (1.7%), and Other (.8%). Participants were accompanied most often by their mother (95%). Median household income was $75,000-$79,999 and 75% of parents had a bachelor’s degree or higher.

### Procedure Overview

Data were collected during one 90-minute laboratory visit. On arrival to the laboratory playroom, parents (one primary caregiver per child) were consented to the study, and children were given a brief, age-appropriate overview of planned tasks. Parents filled out questionnaires and underwent a psychiatric interview about their child. During the lab visit, children engaged in several behavioral and computerized tasks in an adjacent room; including two inhibitory-control tasks, one hot (Delay of Gratification Task; [39]), the other cold (Go/No-Go Zoo Task; [40, 41]). Parents were able to watch their children through a one-way mirror, and in case of upset, the task was shortened and parents provided comfort. The Delay of Gratification task was video-recorded for later coding. The study was approved by the university Institutional Review Board.

### Measures

#### Parent-Reported Self-Regulation.

The Child Behavior Questionnaire (CBQ; [42]) is a parent-report questionnaire that measures several different aspects of temperament and behavior. The CBQ was developed to assess temperament in children ages 3 to 8. This measure has 195 items on Likert scales ranging from 1 (extremely untrue of your child) to 7 (extremely true of your child) and yields 15 sub-scales including measures of self-regulation: Attention Focus, Attention Shifting, Impulsivity, and Inhibitory Control. These CBQ sub-scales do not distinguish between “hot” and “cool” self-regulation, as parents are asked to rate children’s behavior across real-life contexts. The attention focus subscale consists of 9 items and measures a child’s tendency to maintain attentional focus during everyday tasks (e.g., “My child, when drawing or coloring in a book shows strong concentration”). The attention shifting scale consists of 5 items and measures a child’s ability to move attention from one activity to the next (e.g., “My child can easily shift from one activity to another”). The impulsivity scale consists of 13 items and measures children’s tendency to act without thinking (e.g., “Sometimes interrupts others when they are speaking”). The inhibitory control subscale is composed of 13 items and measures children’s ability to regulate their behavior (e.g., “Can lower his/her voice when asked to do so”). Cronbach’s alpha for the current sample were: α = .73 for attention focusing, α = .65 for attention shifting, α = .78 for impulsivity, α = .84 for inhibitory control. The modest Cronbach’s alpha for the attention shifting subscale is consistent with previous reports of reliability for this subscale [13, 43]. An average of the attention shifting and attention focus subscales was used as a measure of parent rated attention. The average of impulsivity (reversed) and inhibitory control were used as a measure of parent rated inhibitory control.

#### Behaviorally Measured Self-Regulation

##### Hot Inhibitory Control.

The Delay of Gratification task (adapted from [39]) was used as a measure of hot inhibitory control. For this task, the child is presented with a desirable snack (M&Ms) and told that they can eat a small pile of M&Ms now or wait until told and eat more M&Ms later. The experimenter leaves the child alone in the room with the M&M’s for 7 minutes. The child has the option of ringing a bell to tell the experimenter to come back and earn the small pile of treats. If the child has not rung the bell or eaten the treat, the experimenter returns after 7 minutes to give the child all of the M&Ms. Delay time was calculated in seconds by measuring the time from when the experimenter left the room until the child either rang the bell or ate the treats. Children who waited the entire 7 minutes were assigned the full delay time (420 seconds).

“Anticipation” of the reward was coded from video recordings of the task. Coders (who were blind to other study measures) completed interval coding in 10-second intervals. In each interval, anticipation was coded as physical contact with the bell/reward (2), visual attention on the bell/reward (1), or no anticipation/attention elsewhere (0). A total anticipation score was calculated by summing the anticipation scores from each interval across the task. For children who did not wait the entire 7 minutes (and thus had less time to focus on the reward), their score was calculated as a ratio of the time waited and transformed to be comparable to children who waited the full 420 seconds. Reliability was established on 20% of cases (ICC = .88).

##### Cool Inhibitory Control.

Participants completed the child-friendly Go/No-Go “Zoo” task to index cool inhibitory control behaviors [40, 41]. In the Zoo task, children are asked to help a zookeeper return loose animals to their cages, except three friendly orangutans who are the zookeeper’s “helpers” and should remain free. Children are asked to put the loose animals back in their cages by pressing a button as quickly as they can every time an animal picture is presented (Go Trials), but to withhold their response each time they see an orangutan (No-Go trials).

Children completed 8 blocks of the task, each including 30 Go trials and 10 No-Go trials for a total of 320 trials. For each trial, a fixation cross was presented for 200 to 300 milliseconds (ms), followed by an animal image presented for 750 ms, and a blank screen for 500 ms. Responses could be made during the animal image and blank screen presentation. Each block consisted of novel sets of animal images, balanced on color, animal type and size. The task was presented using Eprime software (Psychology Software Tools, Inc.: Pittsburg, PA). Before the experimental trials of the Zoo task, children practiced on a set of 12 trials, 3 with orangutans and 9 with other animals and could practice multiple times until they understood the task. Accuracy on No-Go trials (percent correct of total No-Go trials) reflects inhibitory control [44].

#### Psychopathology

##### Dimensional.

Parents completed one of two possible versions of the Child Behavioral Checklist (CBCL) based on child age: CBCL for ages 1.5-5 [45] or CBCL for ages 6-18 [46]. The CBCL is comprised of 99 (ages 1.5 – 5) or 113 (ages 6-18) items that measure aspects of the child’s behavior across the past six months. Items are rated using a three-point rating scale (not true, somewhat or sometimes true, very often or always true) and can be combined to generate internalizing and externalizing symptom composite scores. Based on published norms, T-scores were calculated for internalizing and externalizing scores for use in analyses that included children who received either version of the CBCL (1.5 – 5, 6 – 18). Higher T-scores indicate greater problems.

##### Clinical Diagnoses.

The Schedule for Affective Disorders and Schizophrenia for School-Age Children- Present and Lifetime Version (K-SADS-PL; [47]) is a reliable and valid semi-structured interview that generates DSM-IV Axis I child psychiatric diagnoses. Interviews were conducted by clinical psychology trainees and diagnoses were determined by consensus of the clinical research team (including several of the current authors). Based on the results of the K-SADS, children were either assigned a diagnosis or no diagnosis. Diagnoses were classified into either internalizing (major depressive disorder, dysthymia, panic disorder, post-traumatic stress disorder, separation anxiety disorder, obsessive compulsive disorder, social phobia, agoraphobia, generalized anxiety disorder, specific phobia, adjustment disorder with depression or anxiety, depression not otherwise specified and anxiety not otherwise specified) or externalizing (Oppositional Defiant Disorder, Conduct Disorder, Attention Deficit Hyperactivity Disorder, adjustment disorder with disturbance of conduct, and disruptive behavior disorder, not otherwise specified).

### Missing Data

Of the 120 participants, 16% were missing data on the Go/No-go task, 24% were missing data on the Delay of Gratification task, 3% were missing CBCL data, and 3% were missing CBQ data. Reasons for missing data included child/parent refusal, experimenter error, timing of enrollment, or technical problems. Little’s missing completely at random (MCAR) test was significant (χ^2^=79.5 , df = 53 , p = .01), indicating that data was not missing completely at random. Multiple imputation with applicable predictors was used (25 imputations) and analyses were conducted on the pooled results.

### Data Analysis Plan

Continuous variables were standardized and log transformed for skewness when indicated (no-go accuracy, delay time). Outliers (> 3 SD from mean) were winsorized. Bivariate correlations between study variables were examined. Next, linear regressions were conducted to test measures of self-regulation as predictors of dimensionally-measured psychopathology (CBCL), separately for internalizing and externalizing symptoms. Behavioral measures of self-regulation were entered first, followed by parent-report measures to test which domains of self-regulation contribute unique variance to risk for symptomatology. We chose to enter in this order so as to prevent potentially heightened correlations due to mono-reporter bias between the parent-reported self-regulation and the parent-reported symptomatology from masking relevant associations with behavioral tasks.

For the categorical measure of psychopathology (K-SADS), children with disorders were compared with children without disorders, separately for internalizing and externalizing diagnoses. Given the binary nature of the outcome data, logistic regression analyses were utilized. Again, behavioral measures of self-regulation were entered first, followed by parent-report measures to test which domains of self-regulation contribute unique variance to risk for clinical level disorders.

## Results

### Preliminary Analyses

Bivariate correlations between study variables and descriptive statistics are found in Table 1. Of the 120 children in the study, 28 children met criteria for internalizing disorders and 14 children for externalizing disorders based on K-SADS clinical diagnoses.

### Predicting Dimensional Symptoms

#### Internalizing symptoms.

Full results of the linear regression predicting internalizing symptoms are found in Table 2. In the first step, greater waiting time and greater reward anticipation on the delay of gratification test, a behavioral measure of “hot” inhibitory control, predicted fewer internalizing symptoms. In the second step, delay time and anticipation continued to significantly predict, and additionally, parent-reported attention also uniquely predicted fewer symptoms. By contrast, no-go accuracy on the Zoo task, a behavioral measure of cold inhibitory control, and parent rated inhibitory control did not predict internalizing symptoms.

#### Externalizing symptoms.

The linear regression predicting to externalizing symptoms revealed that greater reward anticipation on the delay of gratification task predicted fewer symptoms at a trend level (*p* = .06) in the first step. In the second step, both parent-reported attention and inhibitory control individually predicted fewer externalizing symptoms, and reward anticipation was no longer a unique predictor. By contrast, wait time on the delay of gratification task and no-go accuracy on the Zoo task did not predict externalizing symptoms (see Table 2).

### Predicting Clinical Disorders

In the logistic regression analyses predicting clinically significant internalizing disorders, greater reward anticipation in the delay task and greater parent-reported attention predicted lower likelihood of having an internalizing disorder. In the second step, however, anticipation became only marginally significant. For externalizing disorders, the only significant predictor was parent-rated attention, which predicted lower likelihood of having an externalizing disorder (Table 3).

## Discussion

The aim of the current study was to examine the associations between self-regulation and internalizing and externalizing symptoms in young children. In particular, we sought to determine whether the subcomponents of attention vs. inhibitory control, as well as hot vs. cool systems would be stronger predictors of internalizing and externalizing symptoms and disorders when measured behaviorally and by parent-report. We measured different domains of self-regulation using multiple methods to help elucidate these relationships, including behaviorally measured cool inhibitory control (computerized go/no-go task), and hot inhibitory control (delay of gratification task), as well as parent-reported attention and inhibitory control.

Our results showed that greater parent-reported attention and behaviorally-measured reward anticipation during hot inhibitory control relate to fewer internalizing *and* externalizing symptoms (at a trend level for reward anticipation) when measured dimensionally. Children whose parent-reports indicated greater attention abilities also had a lower chance of either a clinically significant internalizing or externalizing disorder. This is consistent with some prior research that has demonstrated the importance of attention control for both internalizing and externalizing symptoms (e.g. [11, 48]), but it is in contrast to other research which found attention was more strongly associated with internalizing than externalizing problems [12]. Similarly, children who demonstrated better hot inhibitory control on a behavioral task (longer wait time for a reward) also showed fewer internalizing symptoms, though this measure did not significantly relate to externalizing symptoms or chance of clinically significant disorder. Greater parent-reported inhibitory control related to fewer externalizing symptoms only, consistent with prior work [8, 49].

Further, cool inhibitory control did not relate to either internalizing or externalizing symptom, which is also consistent with some prior research [25] but is in contrast to studies that have found associations between cool inhibitory control and symptoms (e.g. [9, 50]). Nor did we find evidence that children can be “over-regulated” (e.g. [51]) though we did not examine interactions with behavioral inhibition as has been done in past studies (e.g. [13]). It may be that cool inhibitory control is more associated with other outcomes such as academics (as found in [25]) than with psychopathology which is inherently emotional. It is also possible that the absence of findings for cool inhibitory control might be due to the relatively low variability on this measure in our sample. Future studies should examine a battery of hot and cool tasks and multiple outcomes to examine these possibilities.

In general, our results suggest that the greater capacity to direct and sustain attention, indexed via parent-report on child behavior in everyday activities, is robustly associated with lower levels of symptomatology across internalizing and externalizing spectrums. This makes sense in that symptoms of both internalizing (e.g. rumination, hyper-focus on anxiety-inducing stimuli) and externalizing (e.g. short attention span, failure to attend to instructions) disorders feature deficits in attention control. We were not able to test whether behavioral measures of attention relate similarly to psychopathology. Further, as parent-rated attention generally indexes “hot” regulation in everyday situations that are inherently imbued with emotion, it will be important for future research to examine whether “cool” attention may also predict internalizing and externalizing symptoms.

The role of inhibitory control is somewhat more complex. Our results suggest that inhibitory control is generally more important for externalizing than internalizing when indexed via parent-report on child, but becomes relevant for internalizing if indexed behaviorally in a hot context. Strictly cool inhibitory control was not related to either internalizing or externalizing symptom, indicating that the ability to inhibit behavior under emotion-laden constructs is more important for psychopathology risk than exclusively cognitive inhibition. It is noteworthy that inhibitory control indexed in these different ways was differentially related to symptomatology (externalizing, internalizing, and no relation). These measures were also not significantly correlated with each other, which could indicate that they are not all tapping into one “inhibitory control” domain, but are in fact measuring different constructs. This would be consistent with a recent study that also found that different aspects of self-regulation (executive control, delay of gratification, and frustration) are differentially associated with symptomatology and appear to be distinct constructs [50]. It may be that hot and cool regulation may reflect different brain mechanisms. Further research using multiple methods to measure inhibitory control to elucidate these differences is warranted.

We did not find any evidence that either subcomponent or system of self-regulation related differently in predicting clinically significant disorders than in predicting across the spectrum of normal to abnormal symptomatology. This is consistent with the RDoC approach that posits that a linear relationship may exist between the construct of self-regulation and both externalizing and internalizing across the full spectrum of severity. Overall, these results highlight the informativeness of examining subcomponents and different systems of self-regulation rather than simply examining self-regulation or effortful control as one unified construct.

Furthermore, we found that anticipation of the reward on the delay of gratification task was significantly associated with internalizing symptoms and disorders, while it predicted externalizing symptoms at a trend level. Surprisingly, children who showed greater anticipation of the reward (attention to the reward/bell and physical contact with the reward/bell) in the delay of gratification task showed *fewer* symptoms. This was unexpected, as anticipation of the reward is considered a liability in terms of task performance; however, in the context of our study where the majority of children passed the task, this behavioral measure may index *better* inhibitory control for a subset of children. That is, children who still successfully delayed but did not need to use distraction or avoid focus on the reward/bell in order to do so may have better inhibitory control than children who needed to avoid the reward/bell in order to succeed. Alternatively, these might be children with high levels of motivation to resist breaking the rules because they so valued the larger reward associated with task compliance. Responsiveness to reward is another RDoC construct, which has been linked to fewer internalizing symptoms, especially depressive symptoms [52]. Thus, when conceptualized this way, it would make sense that more anticipation of the reward would be associated with fewer internalizing symptoms and disorders. The trend level association with fewer externalizing symptoms might be accounted for by hyperactive children who left the task area and thus had very low scores on anticipation while showing elevated externalizing symptoms. Further research with a large sample of children with greater variability in delay task performance could help elucidate these findings.

### Limitations

There were several limitations of the current study. First, our sample size was relatively small which did not allow for moderation analyses and limited our power, especially in the logistic regression analyses. Second, while we measured self-regulation through multi-method assessment, we were only able to compare hot vs. cool regulation in the domain of inhibitory control, as we did not have behavioral assessments of attention. Future studies should include observer report and behavioral indices across both inhibitory control and attention, as well as across hot vs. cool domains. Additionally, it is important to note that inattention is a prominent symptom of several disorders, thus there is some inherent overlap between self-regulation and psychopathology. However, the fact that parent-rated attention related to both internalizing and externalizing disorders, across two different methodologies, supports the conclusion that the associations found were more than just autocorrelations. Especially convincing is the fact that parent-reported attention was associated with the Achenbach empirically derived scales, which do not include attention related items [46]. Finally, this was a cross sectional study, which limits our ability to infer whether self-regulation leads to increased risk of symptoms and disorders across time or whether they simply co-occur. This is important to consider if self-regulation deficits are to potentially be targeted for intervention to reduce later psychopathology. A longitudinal study of children’s self-regulation and psychopathology is currently underway and will examine these influences across development.

## Implications and Conclusions

Despite these limitations, the current study provides valuable information regarding the association between self-regulation and psychopathology in young children. By elucidating the relationships between different domains of self-regulation and internalizing and externalizing symptomatology, these results can help inform future interventions targeted at specific self-regulation skills. Our findings would suggest that improving children’s ability to focus their attention and to inhibit behavior in emotion-laden contexts and could be beneficial for preventing psychopathology in young children.

## Figures and Tables

**Table 1:** Descriptive statistics and bivariate correlations between study variables (pooled results based on 25 imputations).

	1	2	3	4	5	6	M(SD)	range
1. Snack Delay Wait	1						281.9 (148.8)	0-420
2. Snack Delay Anticipation	−.36***	1					27.8 (13.5)	1-99
3. No-go accuracy	.17	−.12	1				.91 (.15)	.15-1.0
4. Parent-reported Attention	.12	.15	−.07	1			4.4 (.67)	2.7-6.3
5. Parent-reported IC	.13	.06	−.04	.65***	1		.26 (.72)	−1.6-2.0
6. Internalizing Symptoms	−.19^	−.24*	−.03	−.28**	−.11	1	45.6 (10.6)	29-70
7. Externalizing Symptoms	−.08	−.16	−.01	−.43***	−.43***	.60***	46.2 (10.4)	28-79

Note: N=120,

^ p < .10,

* p < .05,

** p <. 01,

*** p < .001.

Means, standard deviations and ranges presented on raw variables. Correlations presented between transformed variables.

**Table 2:** Regression Analysis Predicting symptoms (combined results from 25 imputed data sets)

	Variable	*b*	*SE b*	*t*	*P*
Model 1 Internalizing symptoms					

Step 1					
	Delay Wait time	−1.10	.38	−2.88	.004**
	Delay Anticipation	−.39	.11	−3.48	.001**
	No-Go Accuracy	−.11	.43	−.241	.81
	Constant	−2.07	.89	−2.33	.02*
Step 2					
	Delay Wait Time	−.98	.39	−2.51	.01*
	Delay Anticipation	−.34	.11	−3.05	.002**
	No-Go Accuracy	−.17	.42	−.41	.68
	Parent-rated Attention	−.37	.15	−2.47	.01*
	Parent-rated IC	.15	.13	1.13	.26
	Constant	−1.98	.87	−2.28	.02*

Model 2 Externalizing Symptoms					

Step 1					
	Delay Wait time	−.57	.40	−1.43	.16
	Delay Anticipation	−.23	.12	−1.88	.06^
	No-Go Accuracy	−.003	.46	−.006	.99
	Constant	−.98	.95	−1.04	.30
Step 2					
	Delay Wait Time	−.23	.39	−.61	.55
	Delay Anticipation	−.15	.19	−1.23	.22
	No-Go Accuracy	−.15	.42	−.36	.72
	Parent-rated Attention	−.30	.15	−2.03	.04*
	Parent-rated IC	−.30	.12	−2.4	.02*
	Constant	−.66	.87	−.76	.45

Note:

^ p < .10,

* p < .05,

** p < .01

**Table 3:** Logistic Regression Analysis Predicting disorders (combined results from 25 imputed data sets)

	Variable	*b*	*SE b*	*Odds ratio*	*P*
Model 1 Internalizing symptoms					

Step 1					
	Delay Wait time	−1.21	.93	.30	.19
	Delay Anticipation	−.70	.33	.50	.04*
	No-Go Accuracy	−.89	1.06	.41	.40
	Constant	−4.90	2.27	.007	.03*
Step 2					
	Delay Wait Time	−.69	.98	.50	.48
	Delay Anticipation	−.62	.36	.54	.09^
	No-Go Accuracy	−1.24	1.12	.29	.27
	Parent-rated Attention	−1.05	.43	.35	.02*
	Parent-rated IC	.17	.37	1.18	.64
	Constant	−4.74	2.34	.009	.04*

Model 2 Externalizing Symptoms					

Step 1					
	Delay Wait time	.63	1.27	1.88	.62
	Delay Anticipation	−.18	.42	.83	.66
	No-Go Accuracy	−1.17	1.34	.31	.38
	Constant	−3.02	2.77	.05	.28
Step 2					
	Delay Wait Time	2.81	1.86	16.6	.13
	Delay Anticipation	−.06	.60	.95	.93
	No-Go Accuracy	−2.31	2.01	.10	.25
	Parent-rated Attention	−2.13	.84	.12	.01*
	Parent-rated IC	−.62	.58	.54	.28
	Constant	−2.47	3.92	.08	.53

Note:

^ p < .10,

* p < .05
